# Exploring the Therapeutic Potential of Glucagon-Like Peptide 1 (GLP-1) Receptor Agonists in Polycystic Ovary Syndrome

**DOI:** 10.7759/cureus.73687

**Published:** 2024-11-14

**Authors:** Miis Akel, Aya Ziq, Paul Kaldas, Jad Hamden, Abdul Rahman Omari, Allen Silanee

**Affiliations:** 1 Obstetrics and Gynecology, Nova Southeastern University Dr. Kiran C. Patel College of Osteopathic Medicine, Clearwater, USA; 2 Internal Medicine, Nova Southeastern University Dr. Kiran C. Patel College of Osteopathic Medicine, Clearwater, USA; 3 Physical Medicine and Rehabilitation (PM&amp;R), Nova Southeastern University Dr. Kiran C. Patel College of Osteopathic Medicine, Clearwater, USA; 4 Internal Medicine, St. George's University School of Medicine, Doral, USA; 5 Internal Medicine, Nova Southeastern University Dr. Kiran C. Patel College of Osteopathic Medicine, Davie, USA; 6 Obstetrics and Gynecology, Mount Sinai Medical Center, Miami Beach, USA

**Keywords:** androgens, glp-1 receptor agonists, insulin resistance, obese pcos, pcos and insulin resistance, polycystic ovary syndrome (pcos), semaglutide

## Abstract

Polycystic ovary syndrome (PCOS) is a complex endocrine disorder affecting women of reproductive age and is characterized by hormonal imbalances, insulin resistance, and reproductive dysfunctions. Glucagon-like peptide 1 (GLP-1) receptor agonists (GLP-1 RAs) are primarily used in diabetes management to enhance insulin release by stimulating GLP-1 receptors in the pancreas. The search strategy included randomized trials on the management of PCOS symptoms with GLP-1 RAs alone or in combination with other medications; meta-analyses, literature reviews, or case reports were excluded. This scoping review focuses on 11 articles to explore the therapeutic potential of GLP-1 RAs in PCOS patients. The results show significant reductions in body weight, body mass index (BMI), and waist circumference, alongside improvements in insulin sensitivity in PCOS patients treated with GLP-1 agonists. However, the diverse presentation of patients and treatment approaches was a challenge in understanding the exact mechanisms, long-term effects, and safety profiles of GLP-1 RAs in PCOS patients. This study investigates the promising potential of GLP-1 agonists in managing PCOS-related metabolic and reproductive disturbances.

## Introduction and background

Polycystic ovary syndrome (PCOS) is an endocrine disorder characterized by disrupted menstrual cycles, elevated androgen levels, and the presence of polycystic ovaries, which lead to long-term complications. These include metabolic dysfunction, increased cardiovascular risk, and challenges in fertility, collectively diminishing patient quality of life. Studies show that PCOS affects 4%-20% of women globally [1] though this percentage may vary based on criteria and different populations.

The development of PCOS involves a combination of genetic, hormonal, and environmental factors, with insulin resistance and chronic inflammation being significant contributors to its progression and symptomatology. Treatment approaches have traditionally focused on symptomatic relief using hormonal contraceptives, insulin-sensitizing medications like metformin, and lifestyle changes such as diet and exercise [1]. However, challenges exist in the effectiveness of these treatments and patient adherence. This has led to a search for treatment options to improve outcomes for PCOS patients. Our scoping review particularly explores the use of glucagon-like peptide 1 (GLP-1) receptor agonists (GLP-1 RAs), medications originally intended for treating type 2 diabetes, as possible therapies for PCOS management.

GLP-1 RAs have proven effective in promoting weight loss, enhancing insulin sensitivity, and displaying anti-inflammatory properties [2]. These advantages strongly support their use in managing PCOS. However, despite their benefits, precautions need to be taken due to contraindications like a history of medullary thyroid cancer or pancreatitis, as well as the risk of potential adverse reactions such as nausea, vomiting, diarrhea, constipation, headaches, tachycardia, injection site reactions, pancreatitis, or gastroparesis. It is crucial to evaluate their suitability before use. This review aims to enhance the understanding of how GLP-1 RAs can impact outcomes and discusses their potential strengths and weaknesses within the context of PCOS. Additionally, this review serves to raise awareness regarding GLP-1 RA use in PCOS to improve the quality of care for individuals with PCOS through tailor-made treatment strategies.

## Review

Methods

A systematic search of PubMed with Medline was conducted to identify pertinent studies exploring the therapeutic potential of GLP-1 RAs in PCOS, with a cutoff date of December 18, 2023. The search strategy employed a combination of the keywords "GLP-1 receptor agonists" and "PCOS", aiming to comprehensively capture relevant literature on the subject.

The study selection focused on randomized trials providing insights into managing PCOS symptoms with GLP-1 RAs. The inclusion criteria encompassed studies involving populations diagnosed with PCOS and undergoing GLP-1 RA therapy, either alone or in combination with other medications such as metformin. Studies that did not align with our specific inquiry were excluded and labeled as "not relevant." Systematic reviews, scoping reviews, meta-analyses, and case reports were excluded under the "incorrect study type" category. These study types were excluded to maintain the review’s focus on primary research, specifically randomized trials, which offer direct evidence of treatment efficacy. Reviews and case reports, while valuable for broader context or unique cases, do not provide the controlled, generalizable data necessary for the rigorous assessment of therapeutic interventions in larger populations.

**Figure 1:**
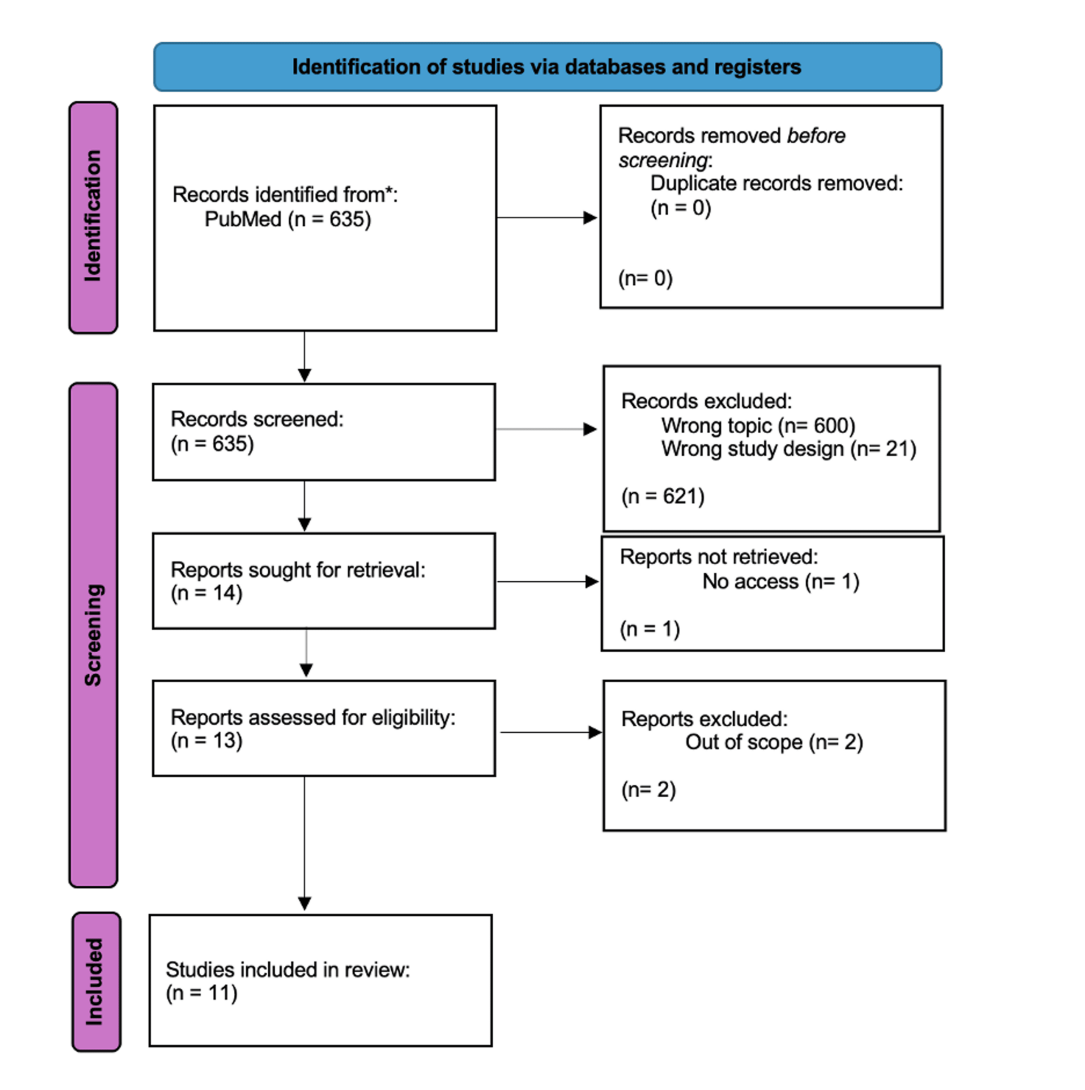
PRISMA study design outlining the article selection process. PRISMA: Preferred Reporting Items for Systematic reviews and Meta-Analyses

The subsequent data extraction process involved a comprehensive analysis of study design, participant characteristics, and treatment outcomes. This meticulous approach ensured a thorough understanding of the methodologies used and the outcomes reported in each study, following a Preferred Reporting Items for Systematic reviews and Meta-Analyses (PRISMA) study design (Figure 1).

Initially, the search yielded 635 publications, which were screened for duplicates, resulting in none. These studies were then screened for relevance, leading to the exclusion of 621 studies due to incorrect study type (21) or irrelevant topics (600). Subsequently, 14 papers were thoroughly reviewed based on the objectives and inclusion/exclusion criteria. From this pool, 11 studies were ultimately selected based on their adherence to the inclusion criteria, study type, and completeness of available data. These selected studies were deemed to provide valuable insights into the use of GLP-1 RAs for PCOS therapy.

Results

Weight Reduction

GLP-1 RAs, including semaglutide, exenatide, and liraglutide, have demonstrated significant benefits in weight reduction for patients with PCOS (Table 1). Multiple studies have reported greater reductions in absolute body weight, body mass index (BMI), and waist circumference, indicative of decreased abdominal adiposity, among those treated with GLP-1 RAs, either alone or in combination with other medications such as metformin [3-13]. These weight loss outcomes appear strongly correlated with improvements in insulin resistance, fertility, and psychological well-being, all of which are key concerns in PCOS management. Notably, a study by Liu et al. [11] found that patients receiving GLP-1 agonists experienced superior weight loss compared to those on metformin alone, contributing to enhanced overall PCOS symptom management.

**Table 1:** Summary of results. This table provides a comprehensive overview of the articles referenced in the results section, including authors, publication year, journal name, study focus, key findings, and relevance to the research topic. OGTT: oral glucose tolerance test; PCOS: polycystic ovary syndrome; ASRM-ESHRE: American Society for Reproductive Medicine-European Society of Human Reproduction and Embryology; BMI: body mass index; LDL: low-density lipoprotein; ELISA: enzyme-linked immunosorbent assay; cIMT: carotid intima-media wall thickness; RHI: reactive hyperemia; GLP-1: glucagon-like peptide 1; SHBG: sex hormone-binding globulin; HOMA-IR: homeostatic model assessment for insulin resistance

Reference	Study design	Data collection	Study aim	Findings	Recommendations	Limitations
[3] Elkind-Hirsch KE, Chappell N, Seidemann E, Storment J, Bellanger D: Exenatide, dapagliflozin, or phentermine/topiramate differentially affect metabolic profiles in polycystic ovary syndrome. J Clin Endocrinol Metab. 2021, 106:3019-33. 10.1210/clinem/dgab408	Randomized controlled trial	Measurements were taken at baseline and 24 weeks, including weight, blood pressure, waist circumference, and body composition via dual-energy X-ray absorptiometry (DXA). OGTTs were conducted	To evaluate the effects of exenatide (EQW), dapagliflozin (DAPA), and phentermine/topiramate (PHEN/TPM) on metabolic parameters, body composition, and sex hormones in obese women with PCOS	EQW/DAPA and PHEN/TPM significantly reduced weight and total body fat. EQW/DAPA was superior in improving mean blood glucose and insulin sensitivity and secretion. All treatments improved fasting glucose, testosterone, free androgen index (FAI), and blood pressure	Dual therapy with EQW/DAPA is recommended over single-agent treatments for clinical and metabolic benefits in obese women with PCOS	Single-blinded design, specific demographic of non-diabetic women aged 18-45, and a relatively short duration of treatment (24 weeks)
[4] Jensterle M, Salamun V, Kocjan T, Vrtacnik Bokal E, Janez A: Short term monotherapy with GLP-1 receptor agonist liraglutide or PDE 4 inhibitor roflumilast is superior to metformin in weight loss in obese PCOS women: a pilot randomized study. J Ovarian Res. 2015, 8:32. 10.1186/s13048-015-0161-3	Prospective randomized open-label trial	n = 45 obese women diagnosed with PCOS according to the ASRM-ESHRE Rotterdam criteria. Participants were randomized into three groups: metformin (1,000 mg BID), liraglutide (1.2 mg QD s.c.), or roflumilast (500 mcg QD). Anthropometric measurements, hormonal and metabolic assessments, and menstrual pattern evaluations were conducted at baseline and at the study endpoint	To evaluate whether liraglutide or roflumilast significantly affects body weight compared to metformin in obese women with PCOS, as well as to assess changes in hormonal and metabolic parameters	Subjects treated with liraglutide and roflumilast experienced significant weight loss compared to metformin, with liraglutide showing superiority in reducing weight, BMI, and waist circumference. Liraglutide also demonstrated improvements in glucose homeostasis, while roflumilast led to reductions in testosterone levels and increased menstrual frequencies	Short-term monotherapy with liraglutide or roflumilast could be considered as an effective option for weight loss in obese PCOS women, with liraglutide being particularly beneficial in improving body composition and glucose metabolism	The study had a relatively small sample size and a short observation period. Lifestyle intervention was introduced but not actively promoted throughout the study. The open-label nature of the study might have influenced participant adherence. Additionally, the study did not have a proper control to draw definitive conclusions solely about the effects of roflumilast on PCOS
[5] Jensterle M, Kravos NA, Goričar K, Janez A: Short-term effectiveness of low dose liraglutide in combination with metformin versus high dose liraglutide alone in treatment of obese PCOS: randomized trial. BMC Endocr Disord. 2017, 17:5. 10.1186/s12902-017-0155-9	Randomized controlled trial	n = 30, the study involved a sample size of 30 obese women with PCOS. Participants underwent anthropometric measurements (weight, BMI, and waist circumference) and assessments of glucose, insulin, and hormone levels at baseline and after 12 weeks. They were also monitored for side effects and adherence to the prescribed medication regimen	The aim was to compare the weight-lowering potential and overall effectiveness of a combination of low-dose liraglutide with metformin against high-dose liraglutide alone in obese women with PCOS	Both treatment regimens resulted in significant weight loss and improvements in metabolic parameters. The liraglutide 3 mg (LIRA3) group experienced greater reductions in weight, BMI, and waist circumference compared to the combination (COMBO) group. COMBO significantly reduced total testosterone levels and was associated with fewer instances of nausea. Both treatments improved glucose homeostasis, but the COMBO treatment also led to a significant decrease in LDL cholesterol	The study recommends considering the combination of low-dose liraglutide and metformin as an effective short-term treatment for obesity in women with PCOS, particularly for its additional benefits in improving androgen profiles and tolerability	The study was limited by its short duration, which does not provide insights into the long-term sustainability of the treatment benefits. The small sample size also limits the generalizability of the findings. Further studies with larger sample sizes and longer follow-up periods are needed to confirm these results
[6] Jensterle Sever M, Kocjan T, Pfeifer M, Kravos NA, Janez A: Short-term combined treatment with liraglutide and metformin leads to significant weight loss in obese women with polycystic ovary syndrome and previous poor response to metformin. Eur J Endocrinol. 2014, 170:451-9. 10.1530/EJE-13-0797	Randomized controlled trial	Forty obese women with PCOS, previously treated with metformin for at least six months, participated in a 12-week open-label, prospective study. They were randomized to metformin alone, liraglutide alone, or combined treatment with both. Lifestyle intervention was not promoted	To evaluate the effectiveness of combined treatment with metformin and the glucagon-like peptide-1 receptor agonist liraglutide on weight loss in obese women with PCOS who had a poor response to metformin alone	The combination therapy was superior to monotherapy with either drug in reducing weight, BMI, and waist circumference. The average weight loss was 6.5 kg in the combination group, 3.8 kg in the liraglutide group, and 1.2 kg in the metformin group. Significant reductions in BMI and waist circumference were also noted in the combination group compared to others	Short-term combined treatment with liraglutide and metformin is recommended for significant weight loss and waist circumference reduction in obese women with PCOS who do not respond adequately to metformin alone	The study's limitations include its short duration, the open-label design, and the small sample size. Nausea was more common in the liraglutide group, although it decreased over time and did not correlate with weight loss
[7] Tao T, Zhang Y, Zhu YC, et al.: Exenatide, metformin, or both for prediabetes in PCOS: a randomized, open-label, parallel-group controlled study. J Clin Endocrinol Metab. 2021, 106:e1420-32. 10.1210/clinem/dgaa692	Randomized, open-label, parallel-group controlled trial	The study included 183 overweight/obese PCOS patients with prediabetes, defined by fasting plasma glucose (5.6-6.9 mmol/L) and/or two-hour postglucose levels (7.8-11.0 mmol/L on OGTT). Participants were treated with exenatide (EX) (10-20 µg daily), metformin (MET) (1,500-2,000 mg daily), or both (COM) for 12 weeks	To evaluate the clinical efficacy of EX, MET, or COM in treating prediabetes in PCOS patients	Impaired glucose tolerance was the dominant prediabetes phenotype. The overall sustained prediabetes remission rate was 50.7%. The COM group had a remission rate of 64%, the EX group had 56%, and the MET group had 32%. EX was associated with superior suppression of two-hour glucose increment in OGTT	EX or COM achieved higher rates of remission of prediabetes among PCOS patients by improving postprandial insulin secretion compared to MET monotherapy	Limitations include the single-center design, relatively small sample size, and short duration of treatment (24 weeks). The study's generalizability may be limited to the Chinese population of PCOS patients with prediabetes. Furthermore, the study did not perform an OGTT at the end of the 12-week treatment period
[8] Jensterle M, Kocjan T, Kravos NA, Pfeifer M, Janez A: Short-term intervention with liraglutide improved eating behavior in obese women with polycystic ovary syndrome. Endocr Res. 2015, 40:133-8. 10.3109/07435800.2014.966385	Randomized controlled trial	n = 36, the study involved obese women with PCOS. Participants' eating behaviors and other relevant parameters were assessed at baseline and after a short-term intervention with liraglutide	The aim was to evaluate the impact of short-term liraglutide treatment on eating behavior in obese women with PCOS	The intervention with liraglutide led to improvements in eating behavior among the participants. Participants reported a reduction in uncontrolled eating and emotional eating. Liraglutide treatment also contributed to significant weight loss	The study suggests that liraglutide can be an effective short-term treatment for improving eating behavior and promoting weight loss in obese women with PCOS	The study's short duration limits the understanding of the long-term effects of liraglutide on eating behavior. The sample size and specifics of the data collected were not detailed in the abstract, indicating a need for further research to confirm these findings
[9] Kahal H, Aburima A, Ungvari T, et al.: The effects of treatment with liraglutide on atherothrombotic risk in obese young women with polycystic ovary syndrome and controls. BMC Endocr Disord. 2015, 15:14. 10.1186/s12902-015-0005-6	Randomized controlled trial	Carotid intima-media wall thickness was measured by B-mode ultrasonography, platelet function by flow cytometry, clot structure/lysis by turbidimetric assays, and endothelial function by ELISA and postischemic reactive hyperemia. Data were presented as mean change (six months – baseline) ± standard deviation	To assess the effects of liraglutide on obesity and cardiovascular risk markers, particularly platelet function, in young obese women with PCOS compared to controls of similar age and weight	At six months, weight was significantly reduced in both groups, with no significant difference between them. Significant reductions in inflammation, endothelial function, and clotting were noted. Basal platelet P-selectin expression was significantly reduced at six months in controls but not in the PCOS group, with no significant changes in cIMT or RHI observed	Liraglutide treatment was associated with weight loss and a significant reduction in atherothrombosis markers. It is recommended as a weight loss medication for simple obesity and may have a potentially beneficial effect on platelet function and atherothrombotic risk	Limitations include the single-center design, relatively small sample size, and short duration of treatment (six months). Additionally, the generalizability of results may be limited to the specific demographic studied (non-diabetic women aged 18-45)
[10] Nylander M, Frøssing S, Clausen HV, Kistorp C, Faber J, Skouby SO: Effects of liraglutide on ovarian dysfunction in polycystic ovary syndrome: a randomized clinical trial. Reprod Biomed Online. 2017, 35:121-7. 10.1016/j.rbmo.2017.03.023	Double-blind, placebo-controlled, randomized trial	The study involved 72 women with PCOS divided into two groups (liraglutide and placebo) in a 2:1 ratio. Measurements included baseline and 26-week follow-up assessments of bleeding pattern, levels of AMH, sex hormones, gonadotrophins, and ovarian morphology	To assess the effects of the GLP-1 analog liraglutide on markers of ovarian dysfunction in overweight women with PCOS	Liraglutide led to significant weight loss (5.2 kg average) and improved bleeding regularity compared to placebo. Liraglutide treatment also resulted in reductions in free testosterone and slight decreases in ovarian volume. SHBG levels increased significantly in the liraglutide group	The study suggests that liraglutide could be an effective treatment for improving ovarian dysfunction in overweight women with PCOS, given its effects on weight loss, hormonal regulation, and bleeding patterns	The study's limitations include its single-center design, short duration (26 weeks), and small sample size, which might limit generalizability. Nausea and constipation were more prevalent in the liraglutide group, indicating the need for monitoring and management of these side effects in the broader application of the treatment
[11] Liu X, Zhang Y, Zheng SY, et al.: Efficacy of exenatide on weight loss, metabolic parameters and pregnancy in overweight/obese polycystic ovary syndrome. Clin Endocrinol (Oxf). 2017, 87:767-74. 10.1111/cen.13454	Randomized controlled trial	n = 136, overweight/obese (OW/OB) women with PCOS, randomized to receive either EXE 10 μg BID (n = 88) or MET 1,000 mg BID (n = 88) for the first 12 weeks. Metabolic parameters were observed at baseline and after 12 weeks. During the second 12 weeks, all participants received MET alone, and the rate of natural pregnancy was tracked	The objective was to evaluate the effects of EXE on reproductive and metabolic functions in OW/OB women with PCOS	After the first 12 weeks, the EXE group had significantly greater weight loss (4.29 ± 1.29 kg vs. 2.28 ± 0.55 kg) and total fat percentage reduction (4.67% ± 0.09% vs. 1.11% ± 0.32%) compared to the MET group. The EXE group showed more significant improvements in insulin resistance (HOMA-IR: 1.30 ± 0.58 vs. 0.59 ± 0.12) and increased menstrual frequency (0.62 ± 0.12 vs. 0.37 ± 0.01). During the second 12 weeks, the natural pregnancy rate was significantly higher in the EXE-treated group compared to the MET-treated group (43.60% vs. 18.70%)	The study suggests that short-term EXE therapy can lead to significant weight loss, reduced central adiposity, improved insulin resistance, and increased menstrual frequency, all of which may contribute to higher pregnancy rates in OW/OB women with PCOS	The study did not provide long-term follow-up data, which is necessary to understand the sustainability of the treatment benefits. The open-label design may introduce bias, and further studies are needed to confirm these findings in a larger population over a longer period
[12] Jensterle M, Kravos NA, Pfeifer M, Kocjan T, Janez A: A 12-week treatment with the long-acting glucagon-like peptide 1 receptor agonist liraglutide leads to significant weight loss in a subset of obese women with newly diagnosed polycystic ovary syndrome. Hormones (Athens). 2015, 14:81-90. 10.1007/BF03401383	Open-label, randomized, prospective study	The study included 32 obese women with newly diagnosed PCOS based on the National Institute of Child Health and Human Development (NICHD) criteria. Participants were randomized to receive either liraglutide (1.2 mg daily) or metformin (1,000 mg twice daily) for 12 weeks. Anthropometric measurements and body composition assessments were conducted using DXA. Blood samples were taken for glucose, insulin, and other metabolic and hormonal analyses before and after treatment. Safety parameters were also assessed	To compare the effects of liraglutide and metformin on body weight and metabolic parameters in obese women with PCOS	Significant reductions in BMI, body weight, waist circumference, and whole-body fat mass were observed in both treatment groups without significant differences between them overall. However, in a subgroup of patients with insulin resistance, severe obesity, and higher metabolic syndrome odds (n = 9), liraglutide was notably more effective than metformin in reducing BMI and other metabolic risk factors	Liraglutide may be more beneficial than metformin for weight loss and metabolic improvements in a specific subset of obese PCOS patients with severe metabolic derangements	The study had a small sample size and a short duration of 12 weeks, which may limit the generalizability of the results. Additionally, the open-label design could introduce biases
[13] Jensterle M, Ferjan S, Ležaič L, Sočan A, Goričar K, Zaletel K, Janez A: Semaglutide delays 4-hour gastric emptying in women with polycystic ovary syndrome and obesity. Diabetes Obes Metab. 2023, 25:975-84. 10.1111/dom.14944	Single-center, randomized, single-blind, placebo-controlled trial	The study involved 20 obese women with PCOS. Gastric emptying (GE) was assessed using ^99m^Tc colloid in a pancake and measured by scintigraphy. Sequential static imaging and dynamic acquisition were performed at baseline and Week 13. Estimation of GE was obtained by repeated imaging of remaining ^99m^Tc activity at fixed time intervals over the course of four hours after ingestion	To evaluate the effect of once-weekly subcutaneous semaglutide 1.0 mg on the late digestive period of GE after ingestion of a standardized solid test meal by using technetium scintigraphy	Semaglutide significantly delayed GE compared to placebo. From baseline to study end, semaglutide increased the estimated retention of gastric contents by 3.5% at one hour, 25.5% at two hours, 38.0% at three hours, and 30.0% at four hours after meal ingestion. Four hours after ingestion, semaglutide retained 37% of solid meal in the stomach compared to no gastric retention in the placebo group. The time taken for half the radiolabeled meal to empty from the stomach was significantly longer in the semaglutide group than in the placebo group (171 vs. 118 minutes)	The study suggests that semaglutide can be effective in managing GE processes in women with PCOS and obesity, potentially aiding in weight management and metabolic control	The study's limitations include its small sample size and short duration. Additionally, the study was conducted at a single center, which may affect the generalizability of the results

Glucose and Insulin Sensitivity

In addition to weight reduction, GLP-1 RAs have demonstrated efficacy in improving glucose levels and insulin sensitivity. Studies consistently reported significant decreases in fasting and mean glucose levels during oral glucose tolerance tests (OGTTs) among patients receiving GLP-1 RAs or combination therapies such as exenatide-dapagliflozin [3,8]. Specific trials highlighted the pronounced reduction in mean blood glucose levels with exenatide-metformin regimens, underscoring the superior efficacy of these combinations in managing hyperglycemia [3,5,7]. Furthermore, fasting insulin sensitivity showed the most substantial improvements with GLP-1 RAs or combinations containing these agonists, particularly in patients with severe insulin resistance [3-5,7-9,11]. This enhanced insulin sensitivity is crucial for mitigating the metabolic risks associated with PCOS.

Endocrine and Hormonal Regulation

While studies exploring GLP-1 RA monotherapy reported no significant changes in endocrine parameters such as total testosterone levels [4,8-11], combinations with metformin demonstrated notable reductions in total testosterone, suggesting a potential role in addressing hyperandrogenism-related symptoms like hirsutism and acne [5-7]. Interestingly, semaglutide showed specific hormonal effects, including reductions in androstenedione, free testosterone, and increased sex hormone-binding globulin (SHBG) levels [13]. On the other hand, liraglutide had a more neutral impact on testosterone levels [12], indicating variability in hormonal responses depending on the specific GLP-1 agonist used.

Menstrual Cycle Regularity and Fertility

GLP-1 RAs also contributed to improvements in menstrual cycle regularity, a critical concern for PCOS patients. Studies found that exenatide, among others, was associated with increased menstrual frequency and a more regular cycle [4,10-11]. These effects were particularly evident when GLP-1 RAs were combined with metformin, where weight loss and hormonal regulation further contributed to cycle normalization and enhanced fertility outcomes. A notable study by Liu et al. [11] demonstrated that patients treated with exenatide had higher natural pregnancy rates and reduced inflammatory markers compared to those receiving metformin. Given the inflammatory nature of PCOS, these findings suggest that reducing inflammation may play a pivotal role in improving ovulation and successful conception.

Discussion

Overview of PCOS Pathophysiology

PCOS presents a complex clinical challenge, encompassing metabolic, endocrine, and reproductive dysregulation. The results of our study, alongside existing literature, emphasize the potential of GLP-1 RAs, such as semaglutide, exenatide, and liraglutide, in addressing various symptoms of PCOS.

Insulin Sensitivity and Weight Reduction

One of the prominent features of PCOS is insulin resistance, often accompanied by obesity and dyslipidemia [14]. Our findings highlight the efficacy of GLP-1 RAs in improving insulin sensitivity and promoting weight loss. Coveleskie et al. conducted a study showcasing that the administration of GLP-1 analogs reduced the sensation of hunger in obese women through direct enhancement in functional connectivity between the left nucleus tractus solitarius and the left thalamus and hypothalamus [15]. These analogs can also act indirectly by inhibiting neuropeptide Y and agouti-related peptides, resulting in heightened satiety and reduced hunger [15]. This effect was observed both in monotherapy and combination therapy settings, with GLP-1 agonists demonstrating superior outcomes compared to alternative weight-loss-inducing medications. Reductions in fasting glucose levels and improvements in glucose tolerance suggest a potential role for GLP-1 agonists in mitigating the metabolic disturbances associated with PCOS [14].

Hormonal Regulation and Androgen Reduction

PCOS is characterized by hormonal imbalances, including elevated androgen levels and disrupted gonadotropin secretion. While the effects of GLP-1 agonists on androgen levels were variable across studies, combination therapies involving metformin showed promising results in reducing total testosterone levels. These findings suggest a potential role for GLP-1 agonists in addressing hyperandrogenism-related symptoms such as hirsutism and acne [2].

Menstrual Cycle Regularity and Fertility

Menstrual irregularities and infertility are common manifestations of PCOS, often attributed to ovulatory dysfunction. Our study demonstrates that GLP-1 RAs, either alone or in combination with metformin, contribute to increased menstrual frequency and improved cycle regularity. This effect appears to be mediated through weight loss and hormonal regulation induced by GLP-1 agonists, potentially enhancing fertility outcomes in PCOS patients. It has been shown that obese women have a lower luteinizing hormone (LH) amplitude and mean serum level of LH, which might result in abnormal ovarian follicular recruitment and development, causing longer follicular phases and indicating ovulatory problems [2]. GLP-1 RAs decrease energy intake and provide adipose tissue reduction, therefore reducing the adverse effects of obesity on the reproductive system in women [2].

Anti-inflammatory Effects

PCOS is associated with chronic low-grade inflammation, contributing to its pathogenesis and associated comorbidities. The observed reduction in inflammatory markers, particularly C-reactive protein (CRP), following treatment with exenatide, emphasizes the anti-inflammatory properties of GLP-1 agonists. The anti-inflammatory effects of GLP-1 agonists may have broader implications for disease management and fertility outcomes as persistent low-grade inflammation is believed to be a trigger in the development of PCOS [16]. Additionally, GLP-1 RAs offer a promising therapeutic option for addressing the complex metabolic and reproductive disturbances characteristic of PCOS. GLP-1 RAs administered orally were found to be equally effective to injected GLP-1 RAs; however, oral GLP-1 RAs depend on the digestive system for absorption and can be affected by gastrointestinal issues like nausea and vomiting [17]. Their efficacy in improving insulin sensitivity, promoting weight loss, regulating menstrual cycles, and reducing inflammatory markers highlights their potential as a comprehensive treatment approach for PCOS [3-13].

Comparing GLP-1 Receptor Agonist Efficacy vs. Current Standard Therapy for PCOS

GLP-1 RAs show significantly higher reductions in BMI, body mass, and body fat percentage in women with PCOS when combined with metformin compared to metformin monotherapy. A recent study has shown that women who were treated with exenatide experienced on average a 4.29 ± 1.29-kg weight loss compared to 2.28 ± 0.55 kg of weight loss in the metformin group (p < 0.001) [18]. In addition, the women who received exenatide experienced a significantly lower android and total fat mass percentage compared to those who received metformin monotherapy (p < 0.001) [18]. Additionally, the menstrual frequency ratio (MFR) was 0.90 ± 0.13 in the exenatide arm compared to 0.68 ± 0.03 in the metformin arm (p < 0.001); this significant increase in MFR was associated with weight reduction [18].

Exenatide therapy significantly reduces insulin resistance in prediabetic women with PCOS compared to metformin monotherapy, aiding in weight loss. Remission rates for prediabetes were 64% in the combination arm and 56% in the exenatide group [18]. Both remission rates were significantly higher than those found in the metformin arm (32%) [18]. Oral contraceptive pills (OCPs) play a crucial role in the management of menstrual irregularities and infertility in women with PCOS. While studies show that GLP-1 RA use can lead to regular menstrual cycles, the exact mechanism or pathophysiology of these effects is not quite understood, emphasizing the need for randomized controlled trials specific to menstrual regularities. A study by Jensterle et al. showed significant improvement in the ovulation rate among all groups (exenatide vs. metformin vs. both), with the highest rate in the combination group and the lowest in the metformin-only group [19]. Furthermore, the improvement in menstrual regularity was significantly correlated with a reduction in body weight, suggesting weight loss to be the primary driving factor behind the reproductive improvement.

Limitations and Future Directions

Despite the promising findings, our study has several limitations. The complexity of PCOS presentation and treatment regimens across studies complicates the interpretation of results [9-11]. Additionally, the majority of included studies were observational or small-scale clinical trials, warranting further investigation through large-scale randomized controlled trials [3,5-7]. Future research should focus on understanding the underlying mechanisms of action of GLP-1 RAs in PCOS pathophysiology, as well as exploring their long-term efficacy and safety profiles. Moreover, comparative studies evaluating the effectiveness of different GLP-1 agonists and combination therapies in diverse PCOS phenotypes are warranted to inform personalized treatment strategies.

## Conclusions

In conclusion, this study highlights the prospective use of GLP-1 RAs in managing PCOS, presenting a versatile approach to address its metabolic and reproductive disturbances. GLP-1 RAs facilitate weight loss, enhance insulin sensitivity, reduce systemic inflammation, and regulate menstrual cycles to treat infertility. However, further research is needed to fully understand their effects and long-term efficacy. Given the complexity of PCOS management, integrating GLP-1 agonists may require a personalized approach for optimization. Overall, exploring GLP-1 RAs offers a promising avenue to enhance the overall care of PCOS patients, potentially providing comprehensive and tailored solutions.
